# Insights into the Human Virome Using CRISPR Spacers from Microbiomes

**DOI:** 10.3390/v10090479

**Published:** 2018-09-07

**Authors:** Claudio Hidalgo-Cantabrana, Rosemary Sanozky-Dawes, Rodolphe Barrangou

**Affiliations:** Department of Food, Bioprocessing and Nutrition Sciences, North Carolina State University, 400 Dan Allen Drive, Campus BOX 7624, Raleigh, NC 27695, USA; chidalg@ncsu.edu (C.H.-C.); rsdawes@ncsu.edu (R.S.-D.)

**Keywords:** CRISPR-Cas systems, CRISPR spacers, virome, phages, microbiome

## Abstract

Due to recent advances in next-generation sequencing over the past decade, our understanding of the human microbiome and its relationship to health and disease has increased dramatically. Yet, our insights into the human virome, and its interplay with important microbes that impact human health, is relatively limited. Prokaryotic and eukaryotic viruses are present throughout the human body, comprising a large and diverse population which influences several niches and impacts our health at various body sites. The presence of prokaryotic viruses like phages, has been documented at many different body sites, with the human gut being the richest ecological niche. Clustered Regularly Interspaced Short Palindromic Repeats (CRISPR) and associated proteins constitute the adaptive immune system of bacteria, which prevents attack by invasive nucleic acid. CRISPR-Cas systems function by uptake and integration of foreign genetic element sequences into the CRISPR array, which constitutes a genomic archive of iterative vaccination events. Consequently, CRISPR spacers can be investigated to reconstruct interplay between viruses and bacteria, and metagenomic sequencing data can be exploited to provide insights into host-phage interactions within a niche. Here, we show how the CRISPR spacer content of commensal and pathogenic bacteria can be used to determine the evidence of their phage exposure. This framework opens new opportunities for investigating host-virus dynamics in metagenomic data, and highlights the need to dedicate more efforts for virome sampling and sequencing.

## 1. Introduction

Over the past decade, many efforts have focused on the study of the human microbiome, encompassing genomic analyses and metagenomic studies, to identify the complex microbial population inhabiting *Homo sapiens*, and influencing health and disease. Corresponding studies have documented the tremendous diversity of the host-associated microbiomes at various body sites, revealed its variability across ethnicities, gender, age, diet, and environmental conditions, and yielded new sequencing technologies and analytical tools that enable more extensive and affordable studies. While some correlations have been established between microbial composition and human health or disease, our understanding and therapeutic pipeline remains relatively primitive [1,2,3]. Accumulation of the vast amount of information has broadened the knowledge of the microbial communities inhabiting our body with high variability between and even within individuals based on the aforementioned variables. This has generated large and complex datasets, obtained via different sequencing platforms. However, there has been a paucity of research effort dedicated to studying the virome composition of our body in comparison to the microbiome studies. An individual’s bacterial community is established soon after birth with increasing diversity during the first years of life. The adult microbiome profile will be influenced by numerous factors such as delivery method, gender, nutrition, antibiotic and medical treatments, among others [4,5,6]. Similarly, the virome of individuals varies and can have an impact on health [7]. In healthy adults, the gut microbiome, one of the most studied and microbial-rich habitats within humans, also contains a sizeable bacteriophage population. The occurrence and diversity of this phage population is stably maintained over time [8,9,10,11] with Orders *Caudovirales* and *Microviridae* being predominant during the first two years of life [12]. In order to identify the non-culturable microbes in a community, metagenomics relies on using targeted amplicons, with highly conserved, specific markers. The 16S ribosomal RNA sequence is used to identify bacteria, and the 18S rRNA or the Internal Transcribed Spacer (ITS) identifies fungi and other micro-eukaryotes [13]. Efforts are underway to develop more comprehensive and precise means to characterize bacterial populations and gain access to strain-level information.

**Figure 1:**
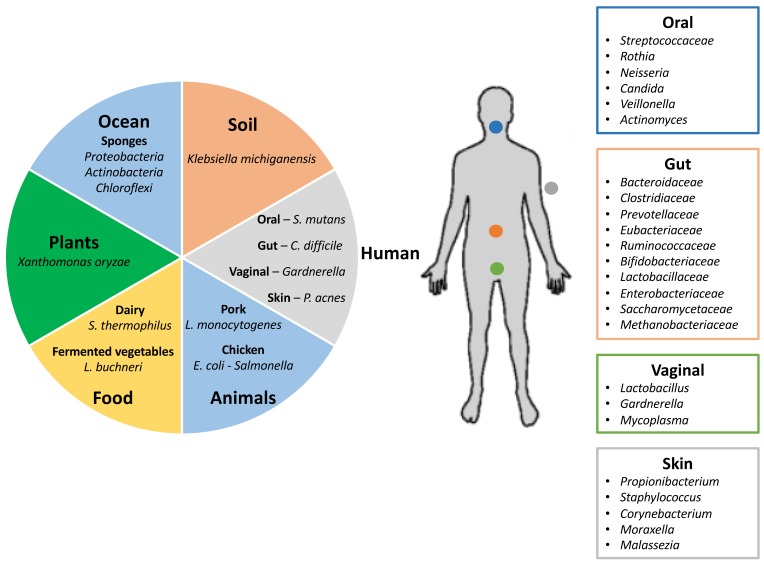
Human microbiome and virome. Different environments showing commensal and pathogenic bacteria that carry Clustered Regularly Interspaced Short Palindromic Repeats (CRISPR)-Cas immune systems as defense mechanisms against invasive nucleic acids from phages and plasmids (**left panel**). Distribution of the human microbiome in the most representative body sites, including some of the species that contain CRISPR-Cas immune systems (**right panel**) (adapted from Lloyd-Price and co-workers [23,24]).

Clustered Regularly Interspaced Short Palindromic Repeats (CRISPR), and CRISPR associated proteins, constitute the adaptive immune system of bacteria and archaea. CRISPR-Cas systems provide defense against invasive nucleic acids, DNA or RNA, from phages and plasmids [14,15]. CRISPR-Cas systems are widespread in nature, and occur in a wide variety of environments. They have been detected in approximately 47% of bacteria and most archaea, in both commensal and pathogenic phyla, genera and species (Figure 1). Within a CRISPR array, the spacers constitute hypervariable regions that are acquired from invasive genetic elements during the host vaccination process. Therefore, they represent a genetic record of phage exposure over time. These spacers can be used as a molecular fingerprint to identify unique strains and therefore as a genotyping tool for commensal bacteria, probiotics [16], as well as pathogenic isolates [17,18,19,20,21,22]. Analyses of the spacer content in CRISPR arrays, from commensal and pathogenic bacteria, and the targeting protospacer, will reveal information about the heterogeneity of viruses present in different environments, such as different body sites, food or environmental samples (Figure 1), as well as the specificity of some viruses to each bacterial or archaeal species. Here, we review the use of CRISPR spacers to elucidate the occurrence and diversity of prokaryotic viruses, found in unique human microbial communities and their body site specificity.

## 2. CRISPR-Cas as Immune System and Vaccination Record

**Figure 2:**
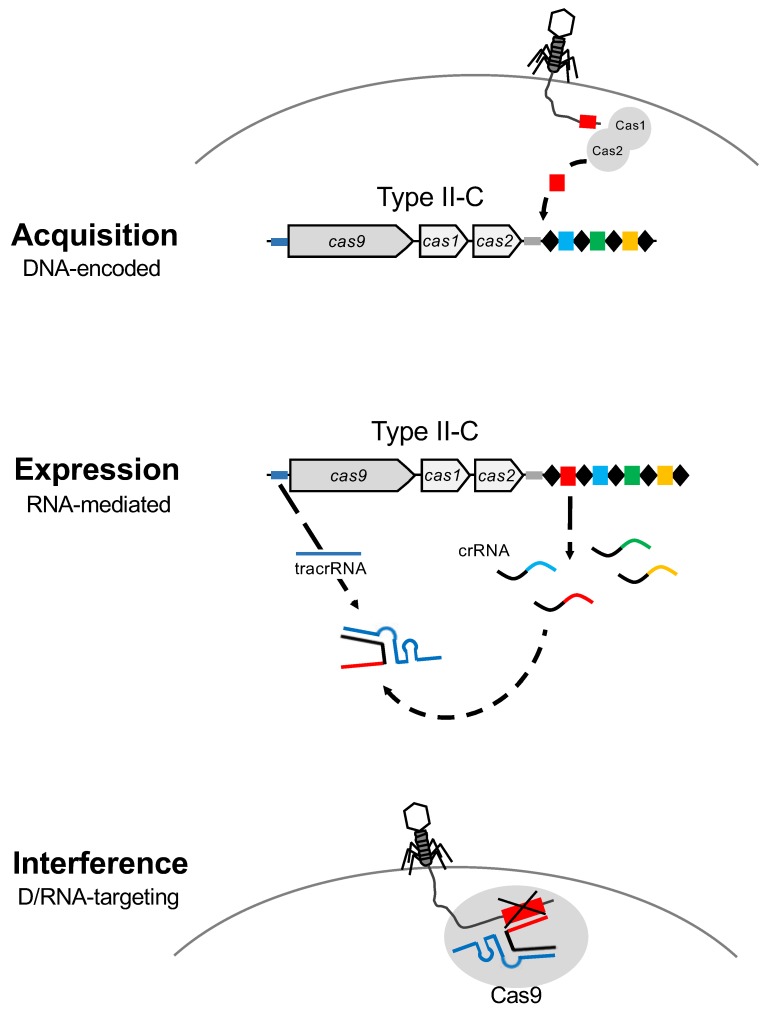
CRISPR-Cas systems: Mode of action. CRISPR-Cas are DNA-encoded, RNA-mediated nucleic acid targeting systems that operate via a three step process. The first step is acquisition, where Cas1 and Cas2 proteins copy and paste a short sequence of the invasive DNA which is added as a new spacer at the 5′ end of the CRISPR array, close to the leader region. During expression, the second step, the CRISPR array is transcribed to generate the mature CRISPR RNAs (crRNA), which are the repeat-spacer pairs, and the trans-activated CRISPR RNA (tracrRNA). Finally, the interference stage is mediated by the duplex crRNA:tracrRNA that drives the Cas9 protein towards the complementary sequence of the spacer in the invasive nucleic acid to bind and cleave DNA after PAM recognition.

CRISPR-Cas systems represent a sequence-specific adaptive defense mechanism in bacteria and archaea against invasive nucleic acids. The CRISPR-Cas locus encompasses a CRISPR array and the *cas* genes that are involved in CRISPR locus maintenance and function (Figure 2). The CRISPR array contains conserved repeat sequences that are separated by spacers, which are hypervariable regions constituting the immunization record of the host. During the spacer acquisition step of the CRISPR mechanism of action, a DNA sequence is copied from the invasive nucleic acid and incorporated as a new spacer in the CRISPR array, at the leader end of the locus, to capture a genetic memory of viral exposure, and immunize the host against the next predatory attack from a virus containing this sequence, either the original invader or a relative thereof (Figure 2). Expression of the CRISPR array leads to the genesis of small CRISPR RNAs (crRNA) that typically contain a portion of a repeat-spacer pair, which act as sequence-specific guides for the Cas effector machinery to detect complementary invasive nucleic acids. Finally, during the interference step, the homology between the spacer portion of the crRNA guide, and the protospacer located within the invasive nucleic acid, drives the Cas effector machinery to specifically recognize, bind, target and cleave complementary sequences, if they are flanked by a protospacer adjacent motif (PAM) [25,26,27,28,29]. Interference hinges on sequence specific cleavage of the target nucleic acid, which is typically lethal for the invasive molecule and prevents completion of the infectious cycle (Figure 2).

The occurrence of diverse CRISPR-Cas systems has previously been found in approximately 90% of archaeal genomes and 46% of bacterial genomes, although it is highly variable depending on the genus or species, with no correlation between CRISPR occurrence and host phylogeny [30]. CRISPR-Cas systems are highly represented in bacterial species that need to overcome continuous exposure to nucleic acid challenges, especially from viruses or bacteriophages that threaten bacterial survival, with thermophiles and extremophiles typically enriched in CRISPR-Cas systems. Noteworthy, lactic acid bacteria widely used in the food supply chain as starter cultures and probiotics are heavily enriched in CRISPR-Cas systems, perhaps reflecting the manufacturing environments they are used in [16,31,32]. Likewise, many human and food borne pathogens also carry CRISPR-Cas systems as a defense mechanism against predatory attack from phages that are present in both food processing and the human body. Therefore, the presence of CRISPR-Cas systems in general, and CRISPR spacers in particular can be used to investigate the interplay between microbiomes and viromes within a particular niche.

In the following sections, we report how the CRISPR spacers extracted from metagenomic data of different human microbiome projects or from deep analysis of certain bacterial strains, have been used to identify and reveal the corresponding virome for various environments.

## 3. Investigating Host-Phage Interactions within Select Body Sites

### 3.1. Gastrointestinal Tract

The gut microbiota contains communities of bacteria, archaea, viruses, fungi, and microbial eukaryotes including yeasts, flagellates, ciliates, protozoa, and (in some geographical locations) intestinal nematodes. However, the majority of viruses are bacteriophages, which are the most abundant biological entity within the gut microbiome [33]; it has been widely studied during recent years using fecal samples. This has been accomplished by performing metagenomic sequencing of microbial communities, and has made gut inhabitants the best characterized and studied to date [5]. Typically, 90% of gut bacteria belong to the *Firmicutes* and *Bacteroidetes phyla* with the other 10% being members of *Proteobacteria*, *Actinobacteria*, *Fusobacteria* and *Verrucomicrobia* [33]. The bacteriophages in the gut are mainly double- and single-stranded DNA viruses (of the *Myoviridae*, *Podoviridae*, *Siphoviridae* and *Microviridae*) that infect *Firmicutes*, *Bacteroidetes*, *Proteobacteria* and *Actinobacteria*. Noteworthy, these viruses can integrate their genomes within the host chromosome as prophages. The gut also contains RNA phages, which are considered transient, of dietary origin and even more poorly characterized than the DNA phages [33]. The generation of large-scale datasets has allowed scientists to not only understand the microbial diversity present in this ecosystem, but also to perform high-throughput analysis of several niches of interest. In particular, the gut microbiome has been analyzed to study the occurrence and diversity of CRISPR-Cas immune systems, as well as determine the CRISPR spacers content to investigate the composition and biological role of the gut virome, and its impact on the microbiome composition and function. For instance, Stern and co-workers were able to identify 991 phages targeted by CRISPR spacers across the gut microbiomes from 124 European subjects [34]. Interestingly, 78% of the detected phages are shared by multiple individuals, some of the phages also being present in stool samples from American and Japanese subjects and from other microbiome projects, suggesting a common virome [34]. Golgeva and co-workers analyzed CRISPR diversity in the human gut metagenomics data from the Human Microbiome Project (HMP), the Healthy Human Gut Metagenomes project from Japan (JPN) and Distal gut metagenomic project [35], using different CRISPR identification algorithms [36]. Despite the fact that some of the CRISPR cassettes identified were not assigned to known bacterial groups, the highest number of contigs harboring CRISPR cassettes were found in *Firmicutes* within three datasets, which is consistent with studies of *Lactobacillus* [32] and *Clostridium* [18], both members of the *Firmicutes* phylum (with occurrence of CRISPR-Cas systems 63% and 100% respectively). The *Actinobacteria* group was the second highest carrier of CRISPR-Cas systems, and specifically the genus *Bifidobacterium* [36]; which is also consistent with other studies of this genus in general [31], and of the *Bifidobacterium longum* species, in particular [16]. The spacer-protospacer analysis displayed differences in the target invasive DNA detected in the three metagenome projects. Overall, the spacers target sequences homologous to *Bacillus* phi29-like phages, and phages that infect *Escherichia*, *Clostridium* spp., *Salmonella*, and several enterobacteria. One of the spacers matched a conserved region of a lambda phage *Ea22* gene, which is present in five different enterobacterial phages (VT2-Sakai, Sf6, Stx1, Stx2, SE1), reflecting a multi-phage resistance provided by a unique spacer [36]. Other spacers matched several unidentified phages isolated from human gut samples, and prophage proteins inserted in bacterial genomes, specifically within *Bifidobacterium.* The protospacers located in bifidobacterial prophages have been previously described [37], with *B. bifidum* LMG11583 one of the most targeted by CRISPR spacers encoded in other bifidobacterial strains [16]. Moreover, CRISPR spacers from the human metagenome projects displayed sequences that match plasmids from *E. coli*, *Salmonella enterica* and *Klebsiella pneumonia*, while other spacers match bifidobacterial transposases or other mobile elements [36]. Another recent study reported CRISPR diversity in the human gut, using gut metagenome datasets from a Chinese population comparing healthy individuals to type-2 diabetic patients [38]. No differences were observed in the occurrence or diversity of CRISPR-Cas systems between the two groups, although higher numbers of CRISPR cassettes were found compared to the previous study performed on European individuals [36]. However, Mangericao and co-workers did not study the CRISPR spacer targets, so no conclusions can be reached regarding the virome composition in Chinese gut, or provide insights into how it differs from European individuals [38].

While CRISPR spacers evolved independently in each bacterial strain, depending on the viral environment, some spacers are conserved among commensal bacterial strains and individuals from different geographic locations, suggesting a common virome in the human gut. Members of the same family (especially twins) share a more similar virome and microbiome, than unrelated individuals [11,12]. The CRISPR spacer analysis of the commensal *B. longum* displayed common spacers among individuals from different locations, that evolved over time with the acquisition of new spacers from childhood to adulthood [16]. The *B. longum* spacers generally matched prophages, as previously mentioned. Other commensal bacteria have been also studied in depth to characterize their CRISPR-Cas systems, e.g., analyses of the spacer targets to predict a functional PAM also demonstrates phage targeting. In this regard, the CRISPR spacer sequences analyzed from several *Lactobacillus gasseri* strains, isolated from healthy human and patient endoscopies, reflect protection against foreign genetic elements such as phages, prophages and plasmids [39].

Interestingly, vertical transmission of phage, from mother to child, through breastfeeding, has been demonstrated for *Bifidobacterium longum* phages 10029 and 10035 as part of the bifidobacterial host [40]. The transmission of phages, as part of the virome, between individuals due to breastfeeding, or the transmission of the virome through fecal microbiota transplantation could have an impact on the final virome population for each individual. This would, therefore, impact the microbiome of each human being, as phages will modulate the microbial community composition due to their host specificity.

In the case of *C. difficile*, a human pathogenic bacterium causing antibiotic-associated nosocomial infections, comparative analysis of the whole genome, including CRISPR-Cas systems has yielded insights into the diversity of strains and their virulence [41]. Type I-B CRISPR-Cas systems were identified in a large data set of 217 genomes, with each system carrying more than one CRISPR array, containing polymorphisms within the spacer content, enabling strain genotyping, as well as determining their phylogenetic relationships [18]. The spacer analyses provided information about the predatory phages that infect *C. difficile* and also the prophages inserted at some chromosomal regions [18,42]. Although less than 40% of the spacers matched a known sequence, every strain presented at least one spacer targeting a *C. difficile* phage, with phiCD27 targeted by a higher number of loci, while phiC2 was targeted by only one strain, suggesting some phage have a wide host range and others may be more strain-specific [42]. Again, the results of these studies are limited by the insufficient number of phages annotated in databases. The increasing interest in alternative therapy to treat the antibiotic resistant *C. difficile* strains has directed isolation of new phage from the Myoviruses group, with wide host-range, including clinical relevant ribotypes [43], and to study the occurrence and evolution of prophage sequences inserted in *C. difficile* chromosomes that could be related to virulence differences between strains [44].

In the case of *Salmonella*, a primary bacterial foodborne and human enteric pathogen, CRISPR-Cas systems have been used for genotyping and studying the diversity among the different serotypes in the species *S. enterica* [17,19,21,22,45,46]. However, despite the extensive use of CRISPR spacers for typing of *Salmonella*, relatively little insight has been provided about *Salmonella*-phage interactions, though 25% of the spacer content provided a protospacer match in databases, including phage, plasmids, prophage and other genome regions unrelated to prophage sequences [21].

### 3.2. Oral

The oral microbiome has also been studied in order to understand the microbial communities dominating the buccal mucosa, the tongue dorsum and the supra-gingival plaque. Results from the expanded Human Oral Microbiome Database (eHOMD) show that 96% of inhabitants of the oral cavity belong to six Phyla: *Firmicutes*, *Actinobacteria*, *Proteobacteria*, *Fusobacteria*, *Bacteroidetes* and *Spirochaetes* [47]. The oral virome plays an important role in health and disease, even more so than the oral microbiome [1,48]. Human oral viruses are mainly bacteriophages with gene-coding functions favoring lysogeny, that impact microbial diversity of the oral cavity [49]. In this regard, the virome comparisons between healthy individuals and patients with periodontal disease displayed a significant difference towards higher abundance of lytic phages in the diseased virome, even though no differences were detected in the microbiome [50]. In the oral microbiome, the CRISPR spacers content, acquisition and evolution in the streptococcal community was studied in four healthy humans, with three sampling points over two months, plus a final sampling after eleven months [51]. Interestingly, constant addition of new spacers was detected at each time point, and high spacer diversity within individuals was found, suggesting the existence of different virus populations in each individual. The spacer target analysis displayed homologous sequences to streptococcal viruses, either *Streptococcus* phages (Cp-1, PH10, PH15 and Ej-1 the most common) or prophages inserted in other streptococcal genomes, as well as *Streptococcus* and *Bacillus* plasmids. These results suggest that the CRISPR-Cas systems in the oral cavity evolve with the acquisition of spacers mainly against *Streptococcus* phages. Some of those spacers are shared by oral and skin microbiome samples, even targeting the same phages previously mentioned [52]. In a different study of the oral microbiome, shotgun metagenomic datasets were used to identified 95,000 contigs containing invasive mobile genetic elements targeted by CRISPR spacers; the oral cavity had more variety in mobile elements, than the stool samples [53]. The protospacers were found in phages, plasmids and integrative and conjugative elements (ICEs), but also in regions of unknown function that should be further explored. Another independent study reported that the distribution of CRISPR-Cas systems are body-site specific and confirmed higher diversity of CRISPR-Cas systems in the oral microbiome than in fecal samples, the vaginal environment or skin microbiome [54]. Regarding the oral microbiome, different samples from the same individual and the same oral site share most of the spacer content, with numbers increasing over time due to acquisition. On the other hand, different oral sites from the same individual shared fewer spacers and different individuals had no common spacers, indicating high variability within individuals, even impacting each person’s oral microbiome composition [54], results that are in accordance with previous studies [51]. The CRISPR spacers are conserved across different oral sites, with few unique spacers to each site, suggesting a common virome in the overall oral cavity, but highly variable within individuals suggesting the virome community is subject-specific [55]. 

### 3.3. Vaginal

The vaginal/urogenital microbiome has been studied in the Human Microbiome Project and in other independent studies, in order to establish a relationship between women’s health and the presence of certain bacterial groups [1,2]. However, the vaginal microbiome is seemingly influenced by ethnicity, age and the menstrual cycle so it is difficult to define a common healthy microbiome, although *Lactobacillus* dominance has been described as desirable in most studies. Lactobacilli seem correlated with a healthy/non-symptomatic status, likely because they are producers of lactic acid, which reduces the pH of the vaginal environment, thereby preventing the survival or growth of acid-sensitive undesirable microorganisms [3,4,56]. Unfortunately, very few studies have focused on the vaginal virome by using metagenomic data to track the occurrence of CRISPR-Cas systems in the vaginal environment and the spacer targets. The CRISPR spacers of *Streptococcus* group B, a genital commensal in healthy women, but a neonatal pathogen, have been used as epidemiological markers to follow vaginal carriage of *Streptococcus* and to understand the evolution against invasive nucleic acids in the vaginal environment [57]. Surprisingly, the analysis of recently acquired spacers and their related targets rarely yielded results from predatory phage attacks, but seemed rather derived from plasmid sequences like *E. faecalis* pLG2 and *S. pseudopneumoniae* pDRPIS7493. Indeed, only one a match to *Streptococcus agalactiae* phage tail gene was identified [57], which is presumably due to the paucity of phage sequences in publicly available databases). However, spacer duplication was detected in 82% of the isolates targeting *Streptococcus dysgalactiae* phage phi3396, which is also related to *Streptococcus pyogenes* [58]. Unfortunately, few studies have used vaginal microbiome metadata to understand the occurrence of CRISPR spacers and elucidate the phage population present in the vaginal environment. 

Regarding *Gardnerella*, a commensal microbe present in the vaginal microbiome of both healthy women and patients with bacterial vaginosis (BV), high occurrence of prophage has been described in *Gardnerella* genomes, suggesting horizontal and vertical transfer that would explain the 400 annotated prophages genes among 39 *Gardnerella* genomes determined to date [59]. The presence of prophage loci and CRISPR Type I systems as defense mechanisms suggest the existence of *Gardnerella*-infecting phages that could be involved in modulating the vaginal microbiome composition, although no bacteriophages have been isolated yet [59]. Indeed, the analyses of CRISPR spacers from *Gardnerella* strains have not provided clues about the invasive nucleic acid attacking this species, due to the absence of homology against databases [60], reflecting the need for increased virome sequencing data from phage and other viruses.

However, several comprehensive studies have been undertaken to understand certain microbial species or strains, analyzing either the CRISPR-Cas systems and spacers or studying the phages that infect those particular hosts. For example, *Lactobacillus jensenii*, a vaginal commensal bacterium, displayed spacers targeting *Lactobacillus* phage Lv-1 [61]. Furthermore, in *L. gasseri*, another vaginal cavity inhabitant, spacer analysis of the vaginal isolate JV-VO3 showed matches against phage KC5a, phage TD1 and also plasmid pHN1 [39]. Limited results have been obtained isolating vaginal phages although seven temperate phages were previously isolated with two of them, phikc005 and phikc007, displaying a broad host range infecting different *Lactobacillus* species [62]. Similarly, 67 phages were isolated from 209 lactobacilli isolates from women in the United States and Turkey, displaying wide host range infectivity of *Lactobacillus* species [63]. These 67 phages were all characterized as temperate phages, capable of infecting most of the lactobacilli vaginal species, with some phages displaying a broad host range, infecting *L. crispatus*, *L. jensenii*, *L. gasseri*, *L. vaginalis* and *L. fermentum* [63]. The 67 phages were all double-stranded DNA and were in four different groups according to the head morphology, size and tail length and contractility; the most representative being phikc21T, phikc12a, phikc39, phikc7a; although no correlation was established among phage type and vaginal health status [63].

In *E. coli*, a ubiquitous bacterium present in many different environments and also, the primary culprit of urinary tract infections in women, the CRISPR-Cas systems have been extensively analyzed. Metagenomic CRISPR spacer-based genotyping allowed the study of phylogenetic diversity and evolution, over time and geographic locations [64,65,66], with incredible conservation of ancient spacers in *E. coli* coming from a forty thousand year-old mammoth [67]. The analyses of the origin of the CRISPR spacers showed low number of protospacer matches to known sequences, again likely due to the shallow viral sequencing performed to date. When protospacer targets were detected, they belong to phage like P2, lambda-like phages [64], phages NJ0, phiEco32, P7, JLK-2012 but also plasmids and prophages [64,65,66,67]. However, the urinary tract *E. coli* isolates displayed fewer numbers of spacers than from fecal isolates, indicating that the human gut is a more phage-rich environment than the vaginal cavity [68]. Interestingly, the urinary tract isolates were more resistant to different antibiotics because resistance is gained by plasmid acquisition and horizontal transfer; this makes sense, since most spacers did not target plasmids [68].

### 3.4. Skin

The skin microbiome presents a wide diversity of bacteria, viruses and fungi, with several individual species associated to disease. About 99% of skin microbiome members belong to one of 4 dominant Phyla: Actinobacteria, Firmicutes, Proteobacteria and Bacteroidetes [69]. The skin microbiome is primarily colonized by *Staphylococcus epidermidis*, *Corynebacterium* and *Propionibacterium*, among others [69]. However, the diversity of microorganisms varies among individuals and skin body sites, being influenced also by genetic factors, diet, medical treatments, hygiene, environmental conditions and life style [70,71]. The skin microbiome is highly altered in certain skin diseases such as psoriasis, dermatitis and rosacea although it remains unclear whether the alteration of the microbiome is the cause or the consequence of the disease [72,73,74]. Moreover, skin microbiome alterations can reflect gut microbiome dysbiosis which are linked to immunological disorders [75,76]. Bacteriophages play an important role in bacterial ecology in their ability to alter microbial populations and spread genetic elements through horizontal transfer [77]. Regarding the skin, *Staphylococcus aureus* (pro)phages are thought to be responsible for the emergence of antibiotic resistance strains, like methicillin-resistant *S. aureus* (MRSA), with seven different phage groups described and associated with different clonal lineages of the *S. aureus* species [78]. However, despite the high number and variety of prophages described for *S. aureus*, CRISPR spacer analyses of all 5000 genomes available at NCBI showed that most of them matched mobile genetic elements, with a few spacers targeting the phage GRCS and other related phages [78]. In another independent study, the CRISPR spacers of *S. aureus* displayed targets against a *lukPV* gene of a phage chromosome, encoding a PVL toxin [79].

The virulent nature of phages and their host specificity can be used to develop phage therapies to reshape the skin microbiome, and selectively eradicate virulent bacterial strains. For *S. aureus*, an encapsulated phage cocktail has been used to kill MRSA strains with an outcome displaying great efficiency in reducing skin infections in rats [80]. Moreover, weaponized phage engineered to express CRISPR arrays have selectively eradicated virulent *S. aureus* strains, repurposing the endogenous CRISPR-Cas systems against their own genome (self-targeting) leading to cell death. This strategy has been employed targeting the antibiotic resistance genes, usually encoded plasmids, to destroy the plasmids, and prevent the spread of plasmid-borne resistance genes [81]. Recently, the endogenous CRISPR-Cas system Type III-A of *S. aureus* was harnessed against the methicillin cassette integrate in the chromosome obtaining a sensitive strain [82].

In *Staphylococcus epidermidis*, a human commensal bacterium, but also a cause of nosocomial infection together with *S. aureus*, the CRISPR-Cas system occurrence is very low, so classical phage therapy can be used as an alternative to kill this bacterium, although some strains carry CRISPR-Cas systems with spacers matching known phages [83]. The analysis of CRISPR spacers in those strains revealed that *S. epidermidis* phage 6ec is widespread, according to the number of spacers targeting this phage, and protospacer targets were also found in a variety of phages, like phage S13’, phage GRCS, philBB-SEP1, phage PH15 and CNPH82, among others [83].

In the case of the opportunistic *Pseudomonas aeruginosa*, CRISPR-Cas systems have been described with high-occurrence in a large data study encompassing more than 600 strains, where CRISPR systems Type I-C, I-E and I-F were detected [84]. When the spacer targets were analyzed, around 26% matched phages, whereas only 1% targeted plasmids like pBS228, pOZ176 and PUM505. Interestingly, 55% of the spacers matched mobile genetic elements, including plasmids like pKLC102. Several spacers in multiple strains targeted the *pilV2* gene of the conjugative type IVb pilus required for functional conjugation-mediated horizontal gene transfer [84]. Also, the protospacer matches allowed identification of several prophages like LESB58 prophage 5, M18 and PA7 prophage RGP78. Indeed, Type I-F CRISPR-Cas systems protect *P. aeruginosa* from phage infections as shown for the wild type phage DMS3 [85] and engineered derivative ones, displaying the relevance of PAM and seed sequence [86]. Moreover, the Type I-F system in *P. aeruginosa* has been described as an active system able to rapidly acquire new spacers against this lytic bacteriophage [87], enabling stable biofilm formation, which can be difficult to eliminate and likely contributes to virulence. However, according to Hoyland-Kroghsbo and co-workers, quorum sensing may be involved in CRISPR-Cas function as a regulatory mechanism, so using anti-quorum-sensing compounds can inhibit CRISPR immune protection in *P. aeruginosa*, making it more suitable for phage therapy [88].

In the case of *Propionibacterium acnes*, Type I-E systems have been described with most of the spacers matching known *P. acnes* phages like PA6, PAD20, PAS50, PAD42, PAD9, PAS40, PAS12 and PAS2, targeting conserved proteins such as head protein, phage DNA primase [89] and mobile genetic elements [90]. The functionality of CRISPR-Cas systems was confirmed in 67 *P. acnes* strains against 15 different phages demonstrating that some of the strains have protection against multiple phages, due to protospacer targeting, but also due to restriction-modification systems present in the bacteria [91]. Diversity and host interaction were also studied using the same phages isolates against other *Propionibacterium* species displaying a broader host range of *P. acnes* phages which can modulate other skin-associated *Propionibacterium* populations. Phage therapy has been proposed to treat acne in order to decrease the population level of *Propionibacterium acnes* [92], and shows potential for treatment of other recalcitrant disorders of the global human microbiome.

## 4. Discussion and Conclusions

**Figure 3:**
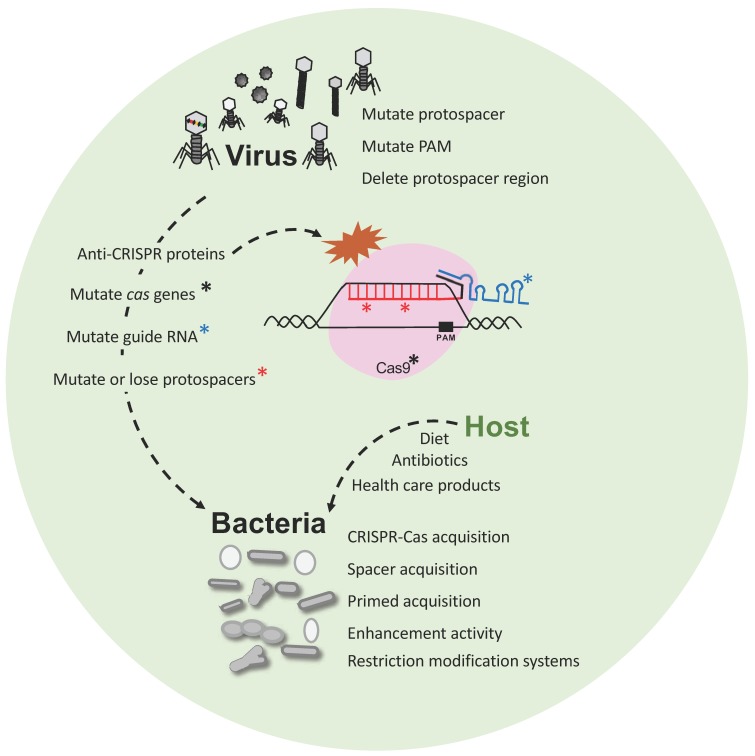
Phage-bacteria dynamics. Interaction and evolution of phage and bacteria developing next generation defense mechanisms, and escape strategies. Bacteria have developed CRISPR-Cas immune systems, among others, that defend against predatory attack from phages whereas the phages also evolve (i) mutating their genome to escape CRISPR targeting; (ii) introducing mutations into CRISPR systems or (iii) developing anti-CRISPR proteins to inactivate the Cas proteins.

Phage-bacteria dynamics within a virome and microbiome population are actively modulated by the interaction between bacterial viruses and their host by continuously evolving mechanisms of attack or escape. Indeed, the host-virus arms race encompasses several defense systems and counter-measures that are impacted by several factors (Figure 3). Phage have evolved with different sizes, shapes and (receptor) tails in order to recognize specific host(s), and perform their lytic or lysogenic cycle, taking advantage of the host metabolism and resources for replication. In this regard, bacteria have evolved and developed several defense systems, notably the CRISPR-Cas adaptive immune system for protection against a predatory attack, so the host can build immunity over time and withstand subsequent exposure to invasive nucleic acid, thanks to the vaccination record, (the CRISPR spacers) (Figure 2). CRISPR-Cas immune systems have also evolved into a wide variety of classes, types and subtypes being effective against DNA and RNA [93,94] to provide diverse and flexible resistance against a wide range of invaders. However, phage-host dynamics are complicated, and encompass continuously evolving escape mechanisms and countermeasures on both sides that drive mutations. Indeed, phages have developed the ability to (i) mutate their own genome to escape to CRISPR targeting, (ii) introduce mutations in the bacteria to inactivate the CRISPR-Cas system or (iii) develop the recently discovered anti-CRISPR proteins that can also inactivate the immune system [95,96] (Figure 3). Importantly, a peculiar case has been described where a phage encodes a complete CRISPR-Cas system on its viral genome. Specifically, *Vibrio cholera* phage ICP1 carries a functional Type I-F CRISPR-Cas system with two different CRISPR arrays containing spacers targeting a highly conserved 18-kilobase region in *V. cholera* chromosome, allowing replication of the phage [97]. This illustrates how phages can co-opt CRISPR-Cas immune systems to actually inhibit host immunity and enable lytic infection by viruses. 

Furthermore, host-phage dynamics is not merely a two-player game, but rather a *menage a trois*, whereby the environment introduces new variables impacting those interactions [33]. Host genetics, mode of delivery, diet and environmental factors, amongst others play an important role in microbiome diversity and composition, although it is still unclear how and to what extent this influences the virome population composition and fluctuation [98]. Diet may not impact the gut and oral virome population directly, and microbial fluctuations, due to dietary changes, can also lead to changes in phage abundance, resulting in modulation of their corresponding bacterial hosts [10]. In the same way, the microbial diversity alterations due to the use of antibiotics, severe medical treatments like chemotherapy or radiotherapy, or even health care products for oral hygiene, skin lotions or feminine hygiene products can likewise lead to the eradication of certain bacterial strains, altering the microbiome at various body sites, thus eliminating the hosts for certain bacteriophages and therefore altering phage populations indirectly.

Virome sampling and analysis will only show virus present at the time of sampling with little information about the historical virome or viruses present in low abundance. Noteworthy, the use of CRISPR spacers represent in this case a powerful tool to analyze the immune record of each body site and the related virome, over time. Analysis of microbiome-associated CRISPR spacers could reveal the virome composition, but more importantly the previous transient viruses (and possibly latent viruses) that have interacted with the habitat and impacted (and may still impact) health. Overall, the scarcity of information obtained from microbiome CRISPR spacers is due to insufficient information about the virome in general, and more importantly, the origin of the virome where the CRISPR spacers were derived from, therefore emphasizing the importance of further studying virome populations to increase the information available in public databases.

Hopefully, the expanded information of the human virome may lead to the discovery of new phage isolates that can be used for therapeutic applications in phage therapy targeting specific bacteria. Having viral sequences available to identify the targets of CRISPR spacers could aid in phage therapy trials, as well as formulating bacteriophage to degrade problematic biofilms in various microbiomes [99]. The overuse of antibiotics has led to the increase of multi-antibiotic resistance in bacteria, therefore, the use of phage therapy has again become an intriguing option in the quest for next-generation antimicrobials that selectively eradicate pathogenic bacteria, without altering the rest of the microbiome, and simultaneously reducing the rise of antibiotic resistance. Indeed, phage therapy has been proposed as a promising alternative that may allow the specific elimination of pathogenic bacteria without altering the microbial community, being especially important in immune-compromised situations like newborns, in whom alteration of the microbiome could have an impact on future health [100,101,102]. There are fewer concerns about using phages in topical application, so phage therapy will most likely be used for bacterial skin disease [98].

The appeal of using phage therapy to treat microbiome disorders is tempered by the limited information available regarding microbiome-associated virome. For example, the healthy gut microbiota, and even the oral microbiome, are thought to be dominated by the difficult to culture Bacteroidetes, and no phage were known in databases until crAssphages were recently discovered metagenomically [103,104]. *Lactobacillus iners* and *Moraxella* species are found in the vaginal and skin microbiomes, respectively, but there is no evidence of bacteriophage for these bacteria in databases that can be used to identify the targets of CRISPR array spacers. 

Overall, virome information deficiencies illustrate the need to dedicate more efforts to the characterization of viruses, and expand our knowledge of bacteriophages, of which more than 90% remain unexplored, encompassing lytic, prophage, phage remnants, Virus Like Particles (VLPs), plasmids (and hosts), RNA and eukaryotic viruses. This would open new avenues for understanding host-phage dynamics, and develop future means to alter microbial compositions to improve human health.
